# Unmasking Type 1 Diabetes in Adults: Insights From Two Cases Revealing Misdiagnosis As Type 2 Diabetes, With Emphasis on Autoimmunity and Continuous Glucose Monitoring

**DOI:** 10.7759/cureus.42459

**Published:** 2023-07-25

**Authors:** Andre E Manov, Sukhjinder Chauhan, Gundip Dhillon, Ashrita Donepudi

**Affiliations:** 1 Internal Medicine, Sunrise Health Graduate Medical Education (GME) Consortium, Las Vegas, USA; 2 Internal Medicine, MountainView Hospital, Las Vegas, USA

**Keywords:** gad-65 antibodies, ia-2 antibodies, zn-t8 antibodies, metformin, basal bolus insulin, diabetic ketoacidosis, hba1c, continuous glucose monitoring (cgm), diabetes mellitus type 2, adult-onset diabetes mellitus type 1

## Abstract

Type 1 diabetes mellitus (T1DM) is often misdiagnosed as type 2 diabetes mellitus (T2DM) in adults, resulting in inadequate treatment and poor disease management. In this report, we present two patients initially misdiagnosed with T2DM for 14 and four years, respectively, leading to complications like diabetic ketoacidosis (DKA). Reevaluation confirmed adult-onset T1DM through antibody tests. Treatment was adjusted to a basal-bolus insulin regimen with the use of continuous glucose monitoring (CGM). The correct diagnosis and CGM implementation significantly improved diabetes mellitus management. This case report emphasizes the importance of mindful diagnosis in adult patients with diabetes mellitus, considering both type 1 and type 2 differentials.

## Introduction

Recent epidemiological data has highlighted that over 50% of all new cases of type 1 diabetes mellitus (T1DM) occur in adults, prompting an investigation into the genetic, immune, and metabolic distinctions between adult-onset and childhood-onset T1DM, though several of these differentiating factors remain poorly understood, presenting obstacles in precise diagnosis and classification [1]. According to data obtained from the United Kingdom (UK) Biobank, more than 40% of individuals with T1DM experience its onset after the age of 30, resulting in frequent misdiagnoses as type 2 diabetes mellitus (T2DM) [2]. The misclassification occurs due to the rarity of adult-onset T1DM, constituting less than 5% of diabetes cases in adulthood, leading healthcare professionals to often assume it to be T2DM [3]. The misdiagnosis carries severe implications, as illustrated in our report, where two patients suffered from diabetic ketoacidosis (DKA) and compromised glucose management.

This case report presents two patients initially misdiagnosed with T2DM, whose clinical profiles revealed the presence of other autoimmune conditions, particularly primary hypothyroidism, prompting further investigation into the possibility of T1DM. Upon subsequent confirmation of T1DM, the patients' oral antidiabetic medications were discontinued, and they were transitioned to an insulin regimen guided by continuous glucose monitoring (CGM) device, resulting in remarkable improvements in glycemic control and significant reductions in glycated hemoglobin (HbA1c) levels. This report emphasizes the significance of comprehensive evaluation and consideration of concurrent autoimmune disorders in cases of patients with adult-onset diabetes presentations to ensure precise diagnosis and suitable management approaches.

## Case presentation

Case 1

A 65-year-old Caucasian female with a medical history of primary hypothyroidism secondary to Hashimoto's thyroiditis, chronic kidney disease (CKD) stage 3b, essential hypertension, chronic hepatitis C, psoriasis, and obesity (with a BMI of 30) presented to our clinic following a recent hospitalization for DKA two weeks ago. Upon discharge from the hospital, the patient was prescribed metformin and basal insulin glargine with a diagnosis of T2DM.

During the patient's initial visit to our clinic, her blood glucose levels at home ranged between 350 and 400 mg/dL, with HbA1c above 14%. Despite adjusting her treatment with up-titration of basal-bolus insulin and metformin for the initial diagnosis of T2DM, her HbA1c remained above 13%, and she reported non-compliance with her diet, insulin regimen, and clinic appointments, attributing her reluctance to cumbersome self-monitoring blood glucose (SMBG). Over the following year, she experienced two hospital admissions for mild and moderately severe DKA due to decompensated diabetes mellitus and pyelonephritis, respectively.

During her second visit to the clinic in late 2021, the patient's frequent episodes of DKA, decompensated diabetes mellitus, and the presence of primary hypothyroidism related to autoimmune Hashimoto's thyroiditis raised suspicion of T1DM, despite her overweight and high BMI. Antibody testing confirmed elevated levels of glutamic acid decarboxylase-65 (GAD-65) antibodies (>250 U/mL), islet antigen-2 (IA-2) antibodies (>350 U/mL), and inc transporter-8 (ZnT8) of 24 U/mL, indicative of autoimmune destruction of pancreatic beta cells and adult-onset T1DM. Insulin antibodies were negative, and her C-peptide level was undetectable, confirming complete autoimmune destruction of her endocrine pancreas (C-peptide <0.1 ng/mL). Primary adrenal insufficiency and autoimmune polyglandular syndrome type 2 (APS-2) were also ruled out as potential contributing factors.

Upon the accurate diagnosis of T1DM, the patient's treatment plan was adjusted to include CGM for optimization of the bolus/basal insulin regimen, thereby addressing challenges related to SMBG and oral antidiabetic medications were discontinued. As a result of this intervention, a significant improvement was observed in her HbA1c levels, decreasing from over 14% to 10.9%.

Case 2

A 52-year-old female with a medical history of T2DM, hypothyroidism, and class III obesity (BMI 40) was admitted to the hospital due to nonspecific abdominal pain and weakness. The hospital workup revealed DKA, leading to her admission to the intensive care unit (ICU) and management with an insulin drip. Despite experiencing two previous DKA episodes in the past two to three years and having hypothyroidism with a possible autoimmune cause, T1DM was not initially considered, and the patient continued to be treated primarily for T2DM. Upon discharge, the patient was prescribed pioglitazone, metformin, and Lantus insulin.

After the discharge from the hospital, during a follow-up visit at our clinic, we explored the possibility of adult-onset T1DM, given the presence of hypothyroidism treated with levothyroxine. Tests revealed elevated levels of GAD-65 antibodies, IA-2 antibodies, and Zn-T8 antibodies, confirming the diagnosis of T1DM with barely detectable C-peptide levels demonstrating complete autoimmune destruction of the pancreatic beta cells. Additionally, the patient had anti-thyroid peroxidase antibodies (TPO) antibodies, indicating Hashimoto's thyroiditis as the autoimmune cause of her hypothyroidism. Primary adrenal insufficiency and APS-2 were ruled out.

After the correct diagnosis of T1DM, the patient's oral antidiabetic medications were discontinued, and she was primarily treated with basal-bolus insulin therapy. The insulin regimen was adjusted using real-time data from CGM. This resulted in a significant improve Hba1c levels from 13% to 7.8%. 

## Discussion

DKA is a condition marked by an imbalance between the lack of insulin and an excess of catecholamines and glucagon. This imbalance leads to metabolic issues such as high blood sugar levels, metabolic acidosis, and the production of ketones [4]. DKA is a severe and avoidable complication of diabetes that poses a life-threatening risk. According to the United States Diabetes Surveillance System (USDSS), there has been a notable rise in hospitalization rates for DKA, particularly among individuals under the age of 45, from 2009 to 2014 [5]. DKA is a life-threatening complication, primarily affecting individuals with T1DM, and poses a significant risk for morbidity and mortality. Moreover, DKA presents a substantial economic burden on individuals, healthcare systems, and payers [6]. Although more commonly observed in patients with T1DM, individuals with T2DM are also at risk, especially during stressful circumstances such as trauma, surgery, or infections [6-7]. 

DKA leads to over 100,000 annual hospital admissions in the United States, accounting for 4-9% of all hospital discharge summaries in patients with T1DM, and necessitating substantial healthcare resources, with one out of every four healthcare dollars spent on direct medical care for adult patients with T1DM, highlighting the importance of effective diabetes management and education to mitigate the burden on the healthcare system [7]. A paradigm shift is necessary to raise awareness about T1DM in adults, as estimates suggest that up to 40% of individuals over 30 years old with T1DM might have been misdiagnosed with T2DM; considering the reduced life expectancy of up to eight years for T1DM compared to three years for T2DM, the clinical and research focus needs to expand to address the challenges of diagnosis and appropriate treatment for T1DM, in addition to the prevailing emphasis on T2DM prevention and treatment in adults [8]. The pathogenesis of T1DM involves T cell-mediated destruction of beta-pancreatic (β-cells), and islet-targeting autoantibodies against specific proteins in β-cells, such as IA-2, GAD-65, Zn-T8 autoantibodies serve as biomarkers of T1DM-associated autoimmunity, detectable months to years before symptom onset, allowing identification and study of at-risk individuals, with the type of autoantibody appearing first influenced by environmental triggers and genetic factors [9].

The main reasons underlying misclassification are multiple and include the lack of awareness among physicians that the onset of T1DM is not limited to children. The majority of older patients have T2DM [10]. Criteria such as BMI and metabolic syndrome suggestive of T2DM can be poor discriminators, especially as rates of obesity in the overall population are increasing [11,12]. The clinical characteristics of adult T1DM are different from those of child-onset T1DM and can resemble the presentation of T2DM, given the slower metabolic progression and risk of metabolic syndrome. Metabolic syndrome occurs in 40% of patients with adult T1DM [11,12]. Also, the prevalence of T2DM is much higher; 90-95% of patients with diabetes mellitus are affected with T2DM vs T1DM, which affects around 5-10% of patients [13]. Due to these biases, physicians frequently forget that reliable markers exist that can help with discrimination between T1DM and T2DM. 

In this report, we presented two patients with adult-onset T1DM. Initially, both were misdiagnosed with T2DM due to their age at diagnosis and lack of weight loss or higher BMI, which was likely secondary to hypothyroidism. Despite receiving treatment with insulin and oral antidiabetic medications, their disease remained uncontrolled, leading to multiple hospital admissions for DKA. During their visit to our clinic, we suspected adult-onset T1DM based on the presence of hypothyroidism likely due to autoimmune etiology and explored the possibility further by checking autoimmune markers such as IA-2, GAD-65, Zn-T8 autoantibodies, and C-peptide levels. The results were positive for GAD-65 and Zn-T8 antibodies in both patients, with one patient also having IA-2 antibodies. Neither patient had detectable C-peptide levels consistent with possible autoimmune destruction of B-cells of the pancreas which are primarily responsible for insulin synthesis and secretion. Furthermore, the patient in Case 2, who was being treated for hypothyroidism, was also found to have anti-TPO antibodies, confirming an autoimmune etiology for her hypothyroidism.

Following the diagnosis of adult-onset T1DM, we discontinued oral antidiabetic medications and treated both patients exclusively with basal-bolus insulin. Additionally, we implemented CGM to monitor their blood glucose levels continuously, leading to significant improvements in blood glucose control. The insulin regimens were adjusted based on real-time CGM data to maintain optimal glucose levels. During the follow-up period of 16 months, the patient in Case 1 was noticed to have a reduction in HbA1c from >14% to 8.6%, while the patient in Case 2 achieved a decrease from >14% to 7.8%. The introduction of real-time CGM also resulted in a reduction in mild and severe hypoglycemia occurrences, with the time spent in the target glucose range (TIR) of 70-180 mg/dL increasing significantly for both patients. These improvements align with the American Diabetes Association (ADA) goals, especially in patients prone to hypoglycemia, where the time spent in the target range should be above 50% [14]. Furthermore, the implementation of CGM positively impacted the patient's eating habits, physical activity, and overall quality of life. This case report emphasizes the importance of accurate diagnosis of T1DM and personalized treatment strategies to optimize diabetes management and improve patients' well-being.

## Conclusions

DKA remains a life-threatening complication of diabetes, primarily affecting individuals with T1DM. The two patients in the present report with adult-onset T1DM were initially misdiagnosed with T2DM, which highlights the critical importance of early and accurate diagnosis. The rising hospitalization rates for DKA, especially among younger individuals, call for urgent attention to address this severe and preventable condition. Healthcare professionals must maintain vigilance in recognizing diverse diabetes presentations and utilize available biomarkers, including IA-2, GAD-65, and Zn-T8 autoantibodies, to distinguish between T1DM and T2DM accurately. Incorporating these autoimmune markers played a pivotal role in confirming the diagnosis of T1DM and enabled the implementation of tailored treatment strategies, including CGM to optimize insulin regimens effectively.

Thus, a paradigm shift toward increasing awareness of T1DM in adults is crucial, aimed at combating misdiagnoses and providing personalized management approaches. By doing so, we can alleviate the burden of DKA on individuals, healthcare systems, and society as a whole, emphasizing the urgency of addressing this condition and its impact on patients' lives. Furthermore, this case report highlights the significance of comprehensive evaluation and consideration of concurrent autoimmune disorders such as hypothyroidism in cases of adult-onset T1DM to ensure precise diagnosis and proper management approaches, ultimately improving patient outcomes and overall healthcare outcomes.
